# Use of the term “romantic love” in the evolutionary social and behavioral sciences

**DOI:** 10.3389/fpsyg.2026.1716892

**Published:** 2026-02-25

**Authors:** Adam Bode

**Affiliations:** School of Archaeology and Anthropology, The Australian National University, Canberra, ACT, Australia

**Keywords:** convention, evolution, love, romantic love, terminology

## 1 Introduction

There is a big problem in psychology and related disciplines related to the inconsistent use of the terminology of love in romantic relationships. The biggest problem is the use of the term “romantic love.” On the one hand, a majority of researchers use the term to describe the motivational state that commonly occurs in the early stages of romantic relationships characterized by particularly intense cognitions, emotions, and behaviors (narrow sense of the term; e.g., Bode and Kushnick, 2021). This aligns with the concept of “passionate love” promoted and used by Elain Hatfield and some others (e.g., Hatfield and Rapson, 1993). On the other hand, some researchers use the term to refer to any love that usually occurs in a romantic relationship, whether it be that state I described, or the less intense love associated with feeling of companionship that characterizes long-term romantic relationships such as marriages (broad sense of the term; Kowal et al., 2025). This dual use of the term leads to bad science as a result of misunderstanding, wasting of time and resources, and false beliefs about love in romantic relationships. There is a convention that the term “romantic love” is used in the narrow sense in the evolutionary social and behavioral science of love that should be adhered to. I am writing this opinion to lay out this convention and to advocate for researchers in the evolutionary social and behavioral sciences to follow this convention. In essence, I argue that the term “romantic love” should be used in the narrow sense, which is in line with convention in the evolutionary social and behavioral sciences (e.g., Bode et al., 2025), the English-language definition (Collins, 2025), the American Psychological Association definition (American Psychological Association, 2018), the general conceptualization in the Triangular Theory of Love (Sternberg, 1986), and the convention in biopsychology (Bode and Kowal, 2023).

### 1.1 The passionate/companionate love distinction

The original classification system used to describe love in romantic relationships was formulated by Elaine Hatfield and her co-authors (e.g., Berscheid and Walster, 1969; Hatfield and Rapson, 1993; Hatfield and Walster, 1985; Walster and Walster, 1978). They distinguished “passionate love” and “companionate love.” Passionate love equates to the state of love commonly associated with the earlier stages of a romantic relationship and particularly strong cognitions, emotions, and behaviors. Companionate love equates to the less intense love felt between individuals in longer-term romantic relationships, such as married couples. The question at hand is whether “romantic love” refers to passionate love or both passionate and companionate love.

### 1.2 Cogent examples of the pitfalls of inconsistent terminology

Problems with methodology is the prototypical negative outcome when it comes to dual use of the term “romantic love.” Not only does it mean that researchers have wasted time and resources, but it creates the false impression in the literature that we know what is either not known or is incorrect. Two recent papers spring to mind that provide good examples of bad science resulting from methodological errors. The first uses the term romantic love in a very broad sense. Han et al. (2024) undertook a bibliographic analysis of the scientific literature to describe the landscape of what they call “romantic love.” In their search terms, they extended the meaning of romantic love so much as to include a search field for “romantic relationship.” This probably resulted from a lack of awareness that the term “romantic love” has a precise meaning, and the authors in turn conflated related concepts associated with romantic relationships. This in effect meant that the analysis considered all studies over the past 10 years that had investigated romantic relationships, and as a result, the findings are not useful in describing the literature on love in romantic relationships. This is problematic because it creates the false impression that there is a sizeable literature on love in romantic relationships and falsely heralds romantic relationship researchers as leaders in the field of romantic love.

A second recent study (Yang et al., 2024) purported to test a theory that the mechanisms associated with romantic love are similar to those involved in addiction by undertaking a meta-analysis of fMRI studies of romantic love and addiction. The theory has always been based upon a narrow definition of the term “romantic love” (see Bode and Kushnick, 2021; Fisher et al., 2016; Wang et al., 2020; Zou et al., 2016). Yang et al. (2024) introduce the theory correctly, drawing on a definition of romantic love that aligns with the narrow sense. However, when it came to the selection of fMRI studies, they included not only fMRI studies of people experiencing “romantic love”—in the early stages of a romantic relationship—but also studies of people in romantic relationships for many years or even decades. This error may have resulted because the authors conducting the study were only superficially familiar with the biopsychological literature on romantic love—one field where the use of the term is notoriously consistent—or the theory. This does not excuse the fact, however, that the corresponding author was a guest editor for a special issue in this Journal on the specific theory they were testing (Zhang et al., 2016) and was therefore aware of the meaning of “romantic love” in the biopsychology literature. The authors concluded that there were few similarities between romantic love and addiction. However, as is clear, this study failed to appropriately test the hypothesis. This is problematic because it creates the perception that the similarities between romantic love and addiction are minimal, despite this not having been tested appropriately. I note that after that article was published, I submitted a letter to the editor at *Neuropscyhologia* highlighting this concern, but it was rejected by the editor.

### 1.3 Scientific terminology

When it comes to scientific terminology, terms should be monosemic, precise, and systematic (Cabré Castellví et al., 1999). Monosemy means that a given term should have only one meaning. This eliminates ambiguity, facilitates exact communication, and enables reproducibility (Cabré Castellví et al., 1999). Precision means that a term is exact and specific. This promotes accuracy of representation, the ability to distinguish concepts, enables fine-grained analysis, and avoids misleading conclusions (Cabré Castellví et al., 1999). Systematicity means that a term falls within a logical, structured, and consistent organization of terms and concepts. This promotes a coherence and structure of knowledge and supports knowledge discovery (Cabré Castellví et al., 1999). For all these reasons, it is necessary for the evolutionary social and behavioral sciences to settle on the correct term to describe the motivational state that commonly occurs at the beginning of a romantic relationship.

## 2 The convention in the evolutionary social and behavioral sciences

A recent book chapter in the International Handbook of Love (Second edition; Bode et al., 2025), summarized the scientific literature on “romantic love” that has used an evolutionary approach. The main contributors to the field (e.g., David Buss; Buss, 2019; Helen Fisher; Fisher, 1998; Garth Fletcher and colleagues; Fletcher et al., 2015; and Adam Bode; Bode and Kushnick, 2021) all use the term “romantic love” in the narrow sense or otherwise choose the term “love” to refer to any love in a romantic relationship. Additional studies have also used the term “romantic love” in the narrow sense (Bode et al., 2025). We are fortunate in the evolutionary social and behavioral science of love to have, to my knowledge, only one instance (Gelbart et al., 2025) of the term “romantic love” being used in the broad sense. As a result, there is much less confusion in this subfield compared to other areas of the psychological sciences. For these reasons, I think it can be said that there is a convention in the evolutionary social and behavioral science of love to use the term “romantic love” in the narrow sense. I believe conventions in scientific terminology should be adhered to unless a valid reason not to exists.

## 3 English language uses the term “romantic love” in the narrow sense

While some of the well-known English language dictionaries (e.g., Oxford, Cambridge, Merriam-Webster) do not have a definition for “romantic love,” some do. For example, Collins Dictionary defines romantic love as “love characterized by romance and involving sexual attraction” (Collins, 2025). Romance and sexual attraction are greatest in passionate love rather than companionate love. This dictionary defines “romantic love” in the narrow sense. This is also the case with Wikipedia (Wikipedia, 2025). A search for the term “romantic love” redirects to a page titled “Romance (love)” Most of the entries refer to “romantic love” and they do so in the narrow sense.

## 4 The American Psychological Association defines “romantic love” in the narrow sense

The American Psychological Association defines romantic love as “a type of love in which intimacy and passion are prominent features. Although the loved party is often idealized, research indicates that the lover's sexual arousal is an especially important component of this type of love” (American Psychological Association, 2018). Passion is associated with the earlier stages of a romantic relationship and passionate love, rather than any love in a romantic relationship. This definition is informed by the Triangular Theory of Love (Sternberg, 1986).

## 5 The Triangular Theory of Love uses the term “romantic love” in the narrow sense

Sternberg's (1986) Triangular Theory of Love is hugely influential in love research. That theory postulates that love can be considered to be constituted by three components, passion, intimacy, and commitment. He asserts that different combinations of these three components result in each of eight different types of love. To my knowledge, there isn't much research confirming these combinations of love components, although it seems evident that passion is indeed higher in the earlier stages of romantic relationships and tends to diminish with time. Some studies have supported the notion that these three components do fluctuate as a function of romantic relationship duration (e.g., Garcia, 1997; Sorokowski et al., 2023) although data from the Romantic Love Survey 2022 (Bode and Kavanagh, 2022) actually indicates that commitment is relatively high in people who self-identify as being “in love.” The theory and its measurement (Kowal et al., 2024; Sternberg, 1997) are robust cross culturally (Sorokowski et al., 2021). Papers are occasionally published which use Sternberg's (1986) terminological framework of love types (e.g., Ribeiro-Gonçalves et al., 2025). This Theory uses the term “romantic love” in the narrow sense.

## 6 Most biopsychologists use the term “romantic love” in the narrow sense

A 2023 review listing all of all biological studies of romantic love up to 2021 (Bode and Kowal, 2023) provides evidence of a strong convention in biopsychology to use the term “romantic love” in the narrow sense. All studies except three were investigating individuals in the early stages of a romantic relationship, and each use of the term “romantic love” referred to the narrow sense of that term. The majority of researchers who have published multiple studies on love, all use the term “romantic love” in the narrow sense. Key among them are Helen Fisher (e.g., Fisher et al., 2005), Arthur Aron (e.g., Aron et al., 2005), Bianca Acevedo (e.g., Acevedo and Aron, 2014), Adam Bode (e.g., Bode and Kushnick, 2021), and Mona Xu (e.g., Xu et al., 2015). There are exceptions among these authors' work, with some occasionally choosing to use the term “passionate love.”

## 7 Discussion

### 7.1 Reasons some authors use the term “romantic love” in the broad sense

The decision to use the term “romantic love” in the broad sense seems to arise from several disparate causes. The first is that many of the authors who do this are not what one would refer to as “love researchers.” As a result, they tend to only be superficially familiar with the literature on love and are prone to making errors of terminology and methodology. I have also noticed that most authors who use the term in the broad sense do not define “romantic love” in their articles, indicating that they have not engaged with the issue of terminology and have simply not given thought to the use of the term. I have also noticed that it is common for non-native English speakers to use the term in the broad sense. It appears that something may be “lost in translation.” Misuse of the term “romantic love” is particularly common among social psychologists. Finally, I must recognize that there are a number of important papers in the domain of love in romantic relationships that have used the term in the broad sense (e.g., Hazan and Shaver, 1987; Kowal et al., 2025), perhaps misguiding those unfamiliar with the literature.

### 7.2 Costs of using the term “romantic love” in the narrow sense

I have pondered the potential costs of adhering to the convention that uses “romantic love” in the narrow sense. I have identified none, except for potentially the opportunity costs of choosing to use the term in the broad sense. I have spoken with multiple love researchers about this topic, and I am utterly unconvinced by any supposed benefits of using the term in a broad sense. These supposed benefits all seem to relate to a more harmonious typology of love which aligns with the term “romantic relationship.”

### 7.3 Benefits of using the term “romantic love” in the narrow sense

The benefits of using the term “romantic love” in the narrow sense are consequential. The first benefit relates to an avoidance of confusion about the meaning of the term “romantic love.” At present, there is little confusion in the evolutionary social and behavioral sciences. Maintaining that convention minimizes confusion in the future. This will achieve the single meaning requirement of scientific terminology (Cabré Castellví et al., 1999). The second substantial benefit is increased precision of the term (Cabré Castellví et al., 1999). An imperfect, but much improved biological definition of romantic love has been proposed in the evolutionary social and behavioral sciences (Bode and Kushnick, 2021). This definition is accompanied by a lengthy review of the biological science of “romantic love” providing exactness to what is being termed “romantic love.” The third substantial benefit relates to embedding the evolutionary social and behavioral science of love within a framework provided by biopsychology. The evolutionary social and behavioral sciences have their roots in evolutionary biology, and draw extensively on biological research (see Bode, 2023). It makes sense that terminology and concepts should be easily transplanted from other biological fields to the evolutionary social and behavioral science of love. This helps the term “romantic love” fall within a logical, structured, and consistent organization of terms and concepts (Cabré Castellví et al., 1999). It also ensures that the evolutionary social and behavioral sciences use the term in line with the English-language and American Psychological Association definitions.

## 8 Conclusion

In this opinion article, I have attempted to raise an issue which I think is of importance to both the science of love and to the evolutionary social and behavioral sciences. The use of the term “romantic love” should be consistent with the principles of scientific terminology. I argue that the correct use of the term is in line with the concept of passionate love (in a narrow sense). This approach is consistent with the convention in the evolutionary social and behavioral sciences, the English-language definition, the American Psychological Association definition, the general definition according to the Triangular Theory of Love, and the convention among biopsychologists. Inconsistency in the use of the term leads to bad science and we should all want to avoid bad science.

The benefits of using the term “romantic love” in the narrow sense far outweigh any supposed costs. As a result, I call on evolutionary social and behavioral scientists to be ruthless in their application of relevant love terminology, and for editors and reviewers at associated journals to enforce the convention. By doing so, we will avoid some of the problems that plague social psychology and can ensure that the evolutionary science of love is of the highest quality possible.
